# Feeling the heat: Investigating interoception and motivation as risk factors for exertional heatstroke

**DOI:** 10.14814/phy2.70529

**Published:** 2025-10-16

**Authors:** Charles Verdonk, Camille Mellier, Keyne Charlot, Arnaud Jouvion, Marion Trousselard, Emmanuel Sagui, Alexandra Malgoyre, Pierre‐Emmanuel Tardo‐Dino

**Affiliations:** ^1^ Department of Neuroscience and Cognitive Science, Unit of Neurophysiology of Stress French Armed Forces Biomedical Research Institute Brétigny‐sur‐Orge France; ^2^ UMR VIFASOM, Université de Paris Paris France; ^3^ Laureate Institute for Brain Research Tulsa USA; ^4^ French Military Health Service Academy Paris France; ^5^ Department of Operational Environments, Unit of Physiology of Exercise and Physical Activities in Extreme Conditions French Armed Forces Biomedical Research Institute Brétigny‐sur‐Orge France; ^6^ Exercise Biology for Performance and Health Laboratory University Evry‐Paris Saclay Evry France; ^7^ French Military Teaching Hospital Laveran Marseille France; ^8^ École de Psychologues Praticiens, Catholic Institute of Paris EA Religion, Culture et société Paris France

**Keywords:** body awareness, exertional heatstroke, interoception, mindfulness, motivation

## Abstract

Exertional heatstroke (EHS) is the most severe form of heat‐related illness, occurring during sport competition or military training. Despite substantial progress in understanding its physiological mechanisms, current evidence suggests the need for broader models that also consider cognitive factors. We propose a cognitive model of EHS and conduct a preliminary empirical validation through a case–control study using self‐report measures. The central hypothesis is that EHS results from a disrupted cost–benefit trade‐off during prolonged physical activity–specifically, an overvaluation of performance‐related benefits due to excessive motivation, coupled with an undervaluation of exertion costs linked to low interoceptive awareness, characterized by disrupted processing of signals related to the body's internal state. Individuals with a history of EHS (cases, *N* = 51) reported significantly lower interoceptive awareness and reduced trait mindfulness compared to controls (*n* = 43). However, no difference was found in global motivation traits between groups. These findings provide initial support for a cognitive model of EHS and suggest that simple self‐report tools may help identify individual vulnerability. Incorporating cognitive dimensions into EHS research could enhance risk stratification and inform new prevention strategies in athletic and military contexts.

## 1 INTRODUCTION

Exertional heatstroke (EHS) is the most serious condition in the spectrum of heat illnesses that can occur during sport competition or physical activity within specific contexts such as military training (Epstein & Yanovich, 2019). The incidence of EHS remains relatively low in sport competitions (Stearns et al., 2017); however, it might become a major health concern in the future because of global warming. Previous research has revealed a large number of risk factors for EHS, including extrinsic factors (e.g., environmental stress such as high temperature or high humidity) as well as intrinsic factors (i.e., individual‐specific factors, such as sleep deprivation or alcohol consumption) (Abriat et al., 2014; Epstein & Yanovich, 2019; Gardner et al., 1996). From a physiological standpoint, EHS is classically described as a non‐compensable heat stress where heat loss does not balance heat gain during a prolonged physical effort (see the Supplementary Introduction: Appendix S1 for a graphical overview of the suspected physiological mechanisms of EHS) (Epstein & Yanovich, 2019; Laitano et al., 2019). Despite substantial progress in understanding the pathophysiology of EHS, which has contributed to the development of preventive strategies for reducing the risk of EHS in sport competitions (Mountjoy et al., 2021; Parsons et al., 2020), the current models are still limited to inform the risk of EHS at the individual level.

Interestingly, several studies have highlighted that cognitive factors (e.g., motivation) can influence the injury risk during sport competition (Ivarsson et al., 2017). For instance, overmotivation has been suggested as a potential risk factor for EHS on the basis of investigation of a relatively small cohort of fatal cases (Rav‐Acha et al., 2004). More recently, Corbett et al. (2017) have investigated the impact of motivation on thermophysiological strain, and they have demonstrated that competition‐induced overmotivation leads to increased thermophysiological cost that may not be perceived (consciously) by the participant (Corbett et al., 2017). This finding suggests that overvaluation of benefits, resulting from overmotivation to succeed, might contribute to the disruption of cost–benefit trade‐off that characterizes adjusted physical effort. The equilibrium between costs and benefits is thought to be a central determinant of behavior. In elite sport, every workload decision—whether made by a coach or an athlete—reflects a trade‐off between performance gains and injury risk. Gabbett et al. (2016) exemplify this using a dynamic metric (the “acute:chronic workload ratio”) that combines short‐term performance benefits, accumulated fatigue, and individual variability to optimize training while minimizing harm (Gabbett et al., 2016).

**FIGURE 1:**
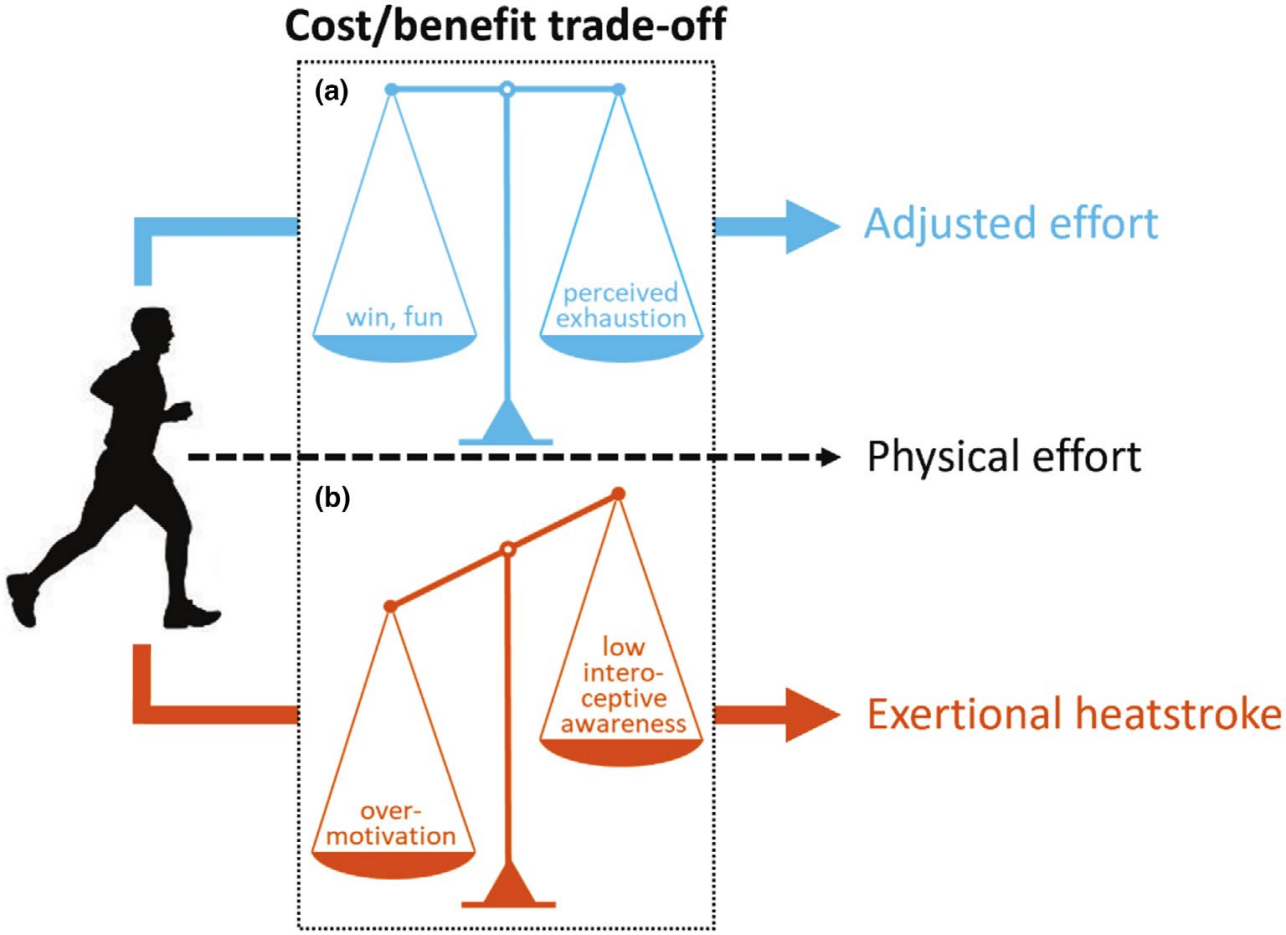
Schematic illustration of the proposed cognitive model for exertional heatstroke. (a) Physical effort in safety conditions relies on a sustained balance between the costs and benefits of effort exertion. (b) We argue that exertional heatstroke is characterized by alteration of the cost/benefit trade‐off. Specifically, the overvaluation of benefits associated with physical effort, as a consequence of overmotivation to succeed, and/or the undervalued costs of effort exertion that result from low body awareness, disrupt the cost/benefit trade‐off and lead individuals to maintain physical effort, which can ultimately lead to exertional heatstroke.

In the present paper, we introduce a model that characterizes EHS by the alteration of cost–benefit trade‐off within the context of prolonged physical effort. Specifically, we propose that EHS could be the consequence of the overvaluation of benefits in combination with the undervaluation of costs associated with physical effort (Figure 1).

Our first assumption is that overmotivation might cause individuals to overvalue benefits that they associate with physical effort (e.g., personal satisfaction, win in the context of competition). Overmotivation has previously been suggested as a potential risk factor for EHS (Epstein & Yanovich, 2019; Rav‐Acha et al., 2004), but it has never been tested empirically. Classical theories of motivation assume that people initiate and persist at behaviors to the extent that they believe the behaviors will meet their needs. In the field of psychology, the term “needs” refers to innate psychological “nutriments” that are essential for ongoing psychological growth, integrity, and well‐being. In the self‐determination theory of motivation (SDT), three psychological needs—for competence, relatedness, and autonomy—are considered essential for understanding motivated behavior (Deci & Ryan, 2000; Ryan & Patrick, 2009). SDT distinguishes intrinsic and extrinsic types of motivation regulating one's behavior. Briefly, the intrinsic motivation is defined as doing an activity because of its inherent satisfaction, for example, for the enjoyment associated with the activity. By contrast, the extrinsic motivation refers to doing an activity for obtaining some outcome separable from the activity per se, for example, for the gain of social reward resulting from win in sport competition. Of note, SDT conceptualizes qualitatively different types of extrinsic motivation as measured with the Global Motivation Scale (see Section 2 for detailed description of the scale). In our model, the motivation is considered as a stable psychological trait rooted in the individual's personality (Scheffer & Heckhausen, 2018), that is, a global motivational orientation, which differs from situational motivation that refers to motivation toward a given activity at a specific point in time, according to the hierarchical model of self‐determined motivation (Vallerand, 2007).

Our second assumption is that sensitivity to bodily signals might influence how individuals self‐regulate their physical activity. Indeed, one can bring awareness to internal body changes caused by physical effort (e.g., heart rate increase, fatigue, and hyperthermia) and ascertain the embodied cost of physical activity. If the cost is assessed as being high, the update of the cost–benefit trade‐off may lead to a decrease in commitment to physical activity, or even a complete stop, to maintain good safety conditions. By contrast, individuals exhibiting low sensitivity to bodily signals may maintain physical effort despite physiological cost, which can ultimately lead to exertional heatstroke. The cognitive construct of body awareness refers to individuals' ability to feel engaged by information coming from within their body and noticing subtle changes (Mehling et al., 2009). From a neurophysiological standpoint, bodily signals continuously provide the brain with a moment‐by‐moment mapping of the body's physiological state, also called interoception, whose integration at higher‐order cortical regions, notably the insula, results in the emergence of interoceptive awareness (Berntson & Khalsa, 2021; Craig, 2002). Interestingly, the insula has been shown to encode cost information during a force task and triggers participants' decision to stop their physical effort (Meyniel et al., 2013). The authors also found that motivation may impact neural processes underpinning effort allocation, specifically by pushing back limits and allowing the body to work closer to exhaustion.

Regarding the potential long‐term clinical applications of our model, we believe that shedding new light on cognitive factors that influence the risk of EHS could ultimately lead to novel preventive interventions. For instance, if low interoceptive awareness characterizes individuals at high risk for EHS, a relevant prevention strategy could be an intervention that enhances interoceptive awareness via effects on the cognitive processing of bodily signals. Contemplative practice, such as mindfulness meditation, has been shown to enhance body awareness by training the mind to pay sustained attention to the body experience and deliberately returning attention to it whenever distracted (Lutz et al., 2015; Treves et al., 2019). It could be argued that the more fully individuals are apprised of what is occurring within their body, the more adaptive and value‐consistent their behaviors are likely to be during physical effort.

To summarize, our cognitive model of EHS suggests that overvaluation of benefits, as a consequence of overmotivation, and undervaluation of costs associated with physical activity, resulting from low interoceptive awareness, could lead individuals to ignore body warning signs of EHS (e.g., hyperthermia, tachycardia, and tachypnea), thus preventing any attempt to self‐regulate physical activity in safety conditions (Figure 1). In the present study, we confronted our model with psychometric data including self‐reported trait motivation, interoceptive awareness, and trait mindfulness, in a cohort of subjects with or without a history of EHS. We predicted that subjects with a history of EHS (cases) would show higher levels of trait motivation, lower interoceptive awareness, and lower levels of trait mindfulness, compared to healthy subjects (controls).

## 2 METHODS

### 2.1 Participants

We recruited 51 patients with a history of EHS (cases) from the French Military Teaching Hospital Laveran (Marseille, France), between 2014 and 2020. Cases were service personnel from conventional combat units of the French Army who have experienced EHS during training or operation, and whose diagnosis and follow‐up care were managed by medical teams from the French Military Health Service. Cases completed self‐report questionnaires during their routine follow‐up visits at the hospital. Data for the present analysis were collected 1,244 ± 199 days (mean ± SEM; range: 800–1,800 days) after the EHS event. There was no exclusion criterion for cases. However, all cases were active‐duty service personnel who regularly underwent medical evaluations to ensure operational readiness, thus limiting significant health comorbidities.

Controls (*n* = 43) were recruited between March 9, 2021 and May 12, 2021, from several French Army units involved in territorial surveillance duties. Like the cases, control participants were career soldiers from conventional operational units. To ensure comparable exertional exposure to cases, all controls had, at some point during their service, completed an 8 km run in battledress—a standard exercise that challenges thermal balance and during which most EHS cases in the French Army occur (Abriat et al., 2014). Controls were matched to cases by morphological characteristics (height and weight), sex, and age. All controls reported no history of EHS, no ongoing medication, and no past or present psychiatric or somatic disorders. Additionally, to ensure relevant operational experience, only soldiers with at least 18 months of service and prior exposure to physical fitness testing and/or operational missions in hot climates were included. Controls completed the self‐report questionnaires in a dedicated experimental session that was planned during their spare time.

### 2.2 Cognitive self‐reported measures

#### 2.2.1 Interoceptive body awareness

The 32‐item Multidimensional Assessment of Interoceptive Awareness (MAIA) questionnaire measures eight facets of body awareness: (1) Noticing: awareness of uncomfortable, comfortable, and neutral body sensations; (2) Not‐distracting: tendency not to be distracted by oneself from sensations of pain or discomfort; (3) Not‐worrying: tendency not to worry with sensations of pain or discomfort; (4) Attention regulation: ability to sustain and control attention to body sensation; (5) Emotional Awareness: awareness of the connection between body sensations and emotional states; (6) Self‐regulation: ability to regulate psychological distress by attention to body sensations; (7) Body listening: actively listens to the body for insight; and (8) Trusting: experiences one own's body as safe and trustworthy (Mehling et al., 2012; Willem et al., 2021). The questionnaire is scored using a six‐point scale, with responses ranging from 0 (never) to 5 (always). For each of the eight subscales, the score was counted by averaging the scores of items belonging to each subscale (items 5, 6, 7, 8, and 9 were reversed). In the present work, the MAIA questionnaire demonstrated acceptable levels of internal consistency in cases (Cronbach alpha = 0.90) and in controls (Cronbach alpha = 0.89).

#### 2.2.2 Self‐determined motivation

The 28‐item Global Motivational Scale (GMS) assesses three types of intrinsic motivation (IM): (1) IM to knowledge: pleasure while learning, exploring, or trying to understand something new, (2) IM to accomplishment: pleasure to accomplish or create something, and (3) IM to stimulation: pleasure to have a stimulating discussion or intense feelings of cognitive pleasure; three types of extrinsic motivation (4) identified regulation: doing something because it matches one's values, (5) introjected regulation: doing something because it is supposed to be good for oneself, and (6) external regulation: doing something in order to have a reward or to avoid punishment; and (7) amotivation: lack of extrinsic or intrinsic motivation (Vallerand et al., 1992). The questionnaire is scored using a seven‐point scale with responses ranging from 1 (not at all) to 7 (totally). For each of the seven subscales assessed by 4 items, the score was counted by averaging the scores of items belonging to each subscale (Vallerand et al., 1992). In the present work, the GMS demonstrated acceptable levels of internal consistency in cases (Cronbach alpha = 0.88) and in controls (Cronbach alpha = 0.93).

#### 2.2.3 Trait mindfulness

The 14‐item Freiburg Mindfulness Inventory (FMI) measures dispositional trait mindfulness by indexing facets of Presence (i.e., being aware of all experiences in the present moment) and Non‐judgmental acceptance (i.e., understanding that things are not necessarily how one wishes them to be) (Trousselard et al., 2010; Walach et al., 2006). This questionnaire is semantically independent from a meditation context, and it is applicable to all population groups, in particular to those with no practice of mindfulness meditation. The questionnaire is scored using a four‐point scale, with responses ranging from 1 (rarely) to 4 (almost always). A total mindfulness score was computed by adding the rating for all items, except for the 13th item, which was reversely scored. In the present work, the FMI demonstrated acceptable levels of internal consistency in cases (Cronbach alpha = 0.80) and in controls (Cronbach alpha = 0.77).

### 2.3 Statistical analyses

Data analyses were performed using JASP (version 0.11.1, https://jasp‐stats.org/). We used both standard statistical tests and Bayesian equivalents to extend insight and guide interpretation of significance (*p* values), according to how likely the alternative hypothesis is versus the null. Indeed, a disadvantage of null hypothesis significance testing is that nonsignificant *p* values (e.g., when reporting no significant difference between cases and controls) cannot be interpreted as support for the null hypothesis (Rouder et al., 2009; Wagenmakers et al., 2018). To circumvent this issue, we calculated the Bayes factor (BF): specifically, we computed the log scale of BF_10_ (noted log(BF_10_)) that can be easily interpreted such that a negative value indicates support for the null hypothesis, whereas a positive value indicates evidence in favor of the alternative hypothesis (see Table S1 for an interpretation scale of log(BF_10_) (Jeffreys, 1961)). Statistical analyses were performed using Mann–Whitney nonparametric tests, as data from cases and controls were not normally distributed. If a significant difference was observed, we computed the effect size using a measure suited to nonparametric analyses: 95% confidence interval of the rank biserial correlation (Glass, 1966). For the Bayesian analyses, we used the default JASP priors that assume a medium effect size on a Cauchy distribution of 0.707 for independent *t*‐tests.

As this is the first study to explore interoception and motivation via self‐report measures in an EHS cohort, no prior data were available to inform an a priori power or sample‐size calculation. However, to ensure that our analyses were nevertheless adequately powered, we conducted post hoc power analyses on significant data (see the Supplementary Methods: Appendix S1).

## 3 RESULTS

### 3.1 Demographic and biometric characteristics

Table 1 summarizes the basic statistics on demographic (age and sex) and biometric (weight, height, and body mass index) measures in cases and controls. Cases and controls did not differ for age (log(BF_10_) = −1.37, suggesting strong evidence for the null hypothesis), sex (log(BF_10_) = −2.03, suggesting extreme evidence for the null hypothesis), weight (log(BF_10_) = −1.50, suggesting very strong evidence for the null hypothesis), height (log(BF_10_) = −1.43, suggesting strong evidence for the null hypothesis), and body mass index (log(BF_10_) = −1.44, suggesting strong evidence for the null hypothesis).

**TABLE 1:** Summary of demographic (age and sex) and biometric (weight, height, and body mass index) data for cases with a history of exertional heatstroke (cases) and controls.

	Cases	Controls	*p* Value^a^	Log(BF_10_)^a^
	(*n* = 51)	(*n* = 43)		
Characteristics				
Age (years), M (SD)	27.80 (6.38)	27.30 (6.48)	0.65	−1.37
Male, *n* (%)	48 (94)	40 (93)	0.83	−2.03
Weight (kg), M (SD)	76.94 (8.41)	77.28 (9.33)	0.89	−1.50
Height (cm), M (SD)	176.24 (7.80)	177.02 (5.17)	0.67	−1.43
Body mass index (kg.cm^−2^), M (SD)	24.79 (2.45)	24.65 (2.76)	0.57	−1.44

Abbreviations: cm, centimeters; kg, kilograms; log(BF_10_), log scale of Bayes factor BF10; M, mean; SD, standard deviation.

^a^ Mann–Whitney nonparametric tests (continuous data) and 𝛘^2^ test (categorical data).

### 3.2 Interoceptive body awareness

**FIGURE 2:**
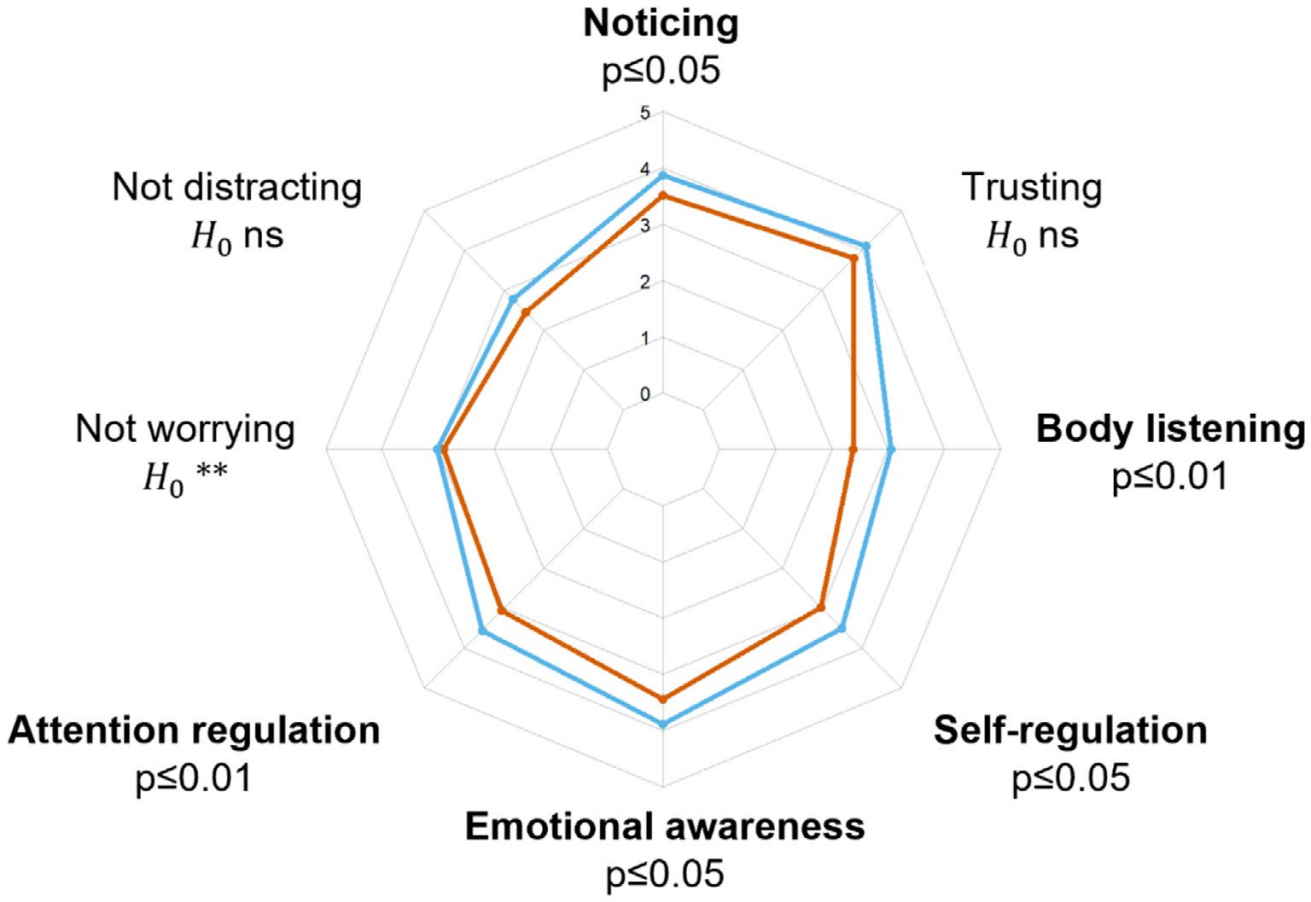
Graphical comparison of cases with a history of exertional heatstroke (orange line) and controls (blue line) with respect to eight facets of interoceptive body awareness, as assessed with the 32‐item Multidimensional Assessment of Interoceptive Awareness questionnaire. Statistically significant group differences are shown in bold. $H_{0}$ ns, anecdotal evidence for the null hypothesis; $H_{0}$**, strong evidence for the null hypothesis.

Five dimensions of interoceptive body awareness were significantly lower in cases compared to controls: Body listening (Mann–Whitney U (U) = 717, *p* ≤ 0.01, 95% confidence interval (CI) of rank‐biserial correlation (rbs) = [0.12–0.54]), Attention regulation (U = 730, *p* ≤ 0.01, 95% CI of rbs = [0.11–0.53]), Emotional awareness (U = 767, *p* ≤ 0.05, 95% CI of rbs = [0.07–0.50]), Self‐regulation (U = 780, *p* ≤ 0.05, 95% CI of rbs = [0.06–0.49]), and Noticing (U = 839, *p* ≤ 0.05, 95% CI rbs = [0.003–0.44]). Furthermore, cases and controls did not differ for three dimensions of interoceptive body awareness: Not‐worrying (log(BF_10_) = −1.36, suggesting strong evidence for the null hypothesis), Trusting (log(BF_10_) = −0.39, suggesting anecdotal evidence for the null hypothesis), and Not‐distracting (log(BF_10_) = −0.20, suggesting anecdotal evidence for the null hypothesis). Figure 2 summarizes how cases and controls differ in terms of self‐reported interoceptive body awareness. Descriptive statistics of self‐reported interoceptive body awareness in cases and controls are reported in Table S2. All significant dimensions of interoception (Body listening, Attention regulation, Emotional awareness, Self‐regulation, and Noticing) produced effect sizes in the medium‐to‐large range, with achieved power between 0.79 and 0.94 (see Table S5), indicating sufficient sensitivity to detect the reported group differences.

### 3.3 Self‐determined motivation

**FIGURE 3:**
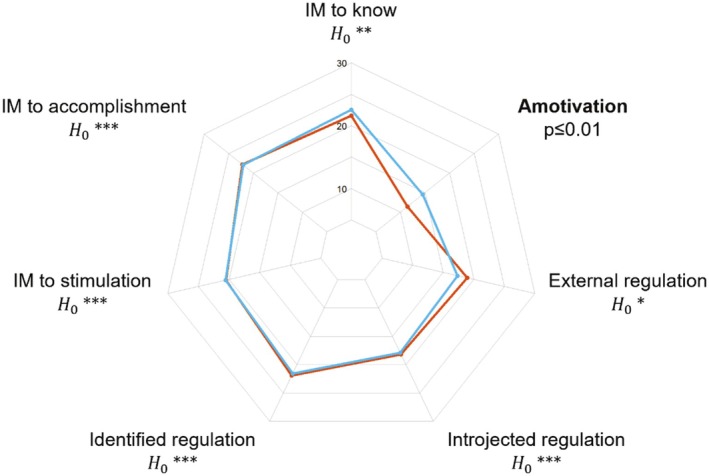
Graphical comparison of cases with a history of exertional heatstroke (orange line) and controls (blue line) with respect to seven factors of self‐determined motivation, as assessed with the Global Motivation Scale. The sole statistically significant group difference is indicated in bold. $H_{0}$*, moderate evidence for the null hypothesis; $H_{0}$**, strong evidence for the null hypothesis; $H_{0}$***, very strong evidence for the null hypothesis.

Cases and controls did not differ for almost all factors of motivation: IM to accomplishment (log(BF_10_) = −1.50, suggesting very strong evidence for the null hypothesis), Introjected regulation (log(BF_10_) = −1.53, suggesting very strong evidence for the null hypothesis), Identified regulation (log(BF_10_) = −1.53, suggesting very strong evidence for the null hypothesis), IM to stimulation (log(BF_10_) = −1.51, suggesting very strong evidence for the null hypothesis), IM to know (log(BF_10_) = −1.21, suggesting strong evidence for the null hypothesis), and External regulation (log(BF_10_) = −0.90, suggesting moderate evidence for the null hypothesis). Only the factor Amotivation was significantly lower in cases compared to controls (U = 729, *p* ≤ 0.01, 95% CI of rbs = [0.11–0.53]). Figure 3 summarizes similarities between cases and controls in terms of self‐determined motivation. Descriptive statistics of self‐determined motivation in cases and controls are reported in Table S3. The motivation dimension “Amotivation” demonstrated a large effect size and achieved power of 0.93 (see Table S5), confirming sufficient sensitivity to detect the observed group difference.

### 3.4 Trait mindfulness

**FIGURE 4:**
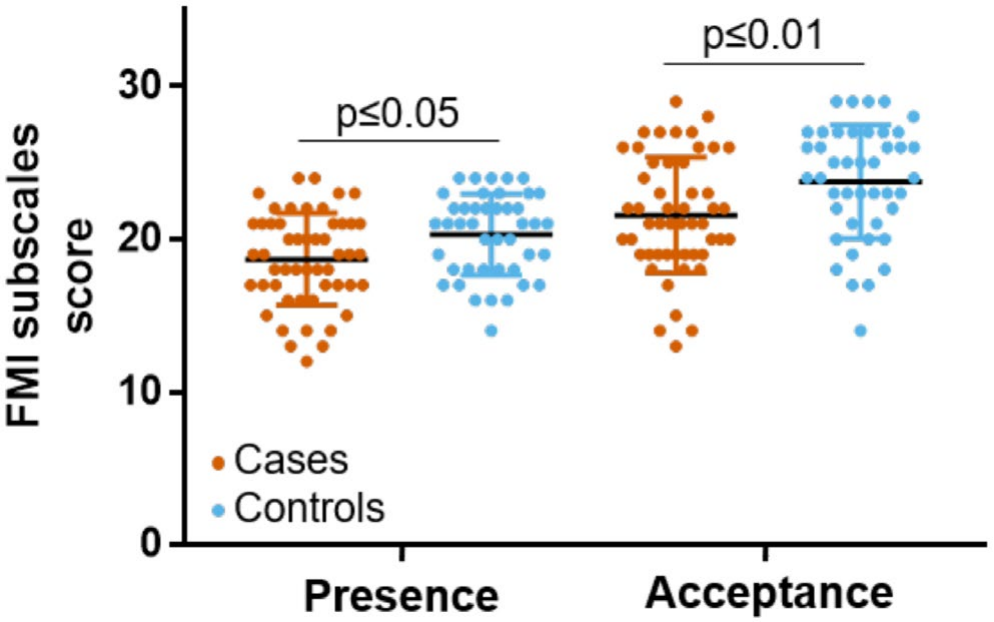
Significant group differences were found between cases with a history of exertional heatstroke (in orange) and controls (in blue) for mindfulness dimensions of Presence and Acceptance, as assessed with the Freiburg Mindfulness Inventory (FMI).

Cases showed lower scores for the two mindfulness dimensions that are assessed with the FMI, relative to controls: Presence (U = 761, *p* ≤ 0.05, 95% CI of rbs = [0.08–0.50]), and Acceptation (U = 730, *p* ≤ 0.01, 95% CI of rbs = [0.11–0.52]) (Figure 4). Descriptive statistics of trait mindfulness in cases and controls are reported in Table S4.

## 4 DISCUSSION

### 4.1 General discussion

This paper introduces a testable model that explores cognitive factors influencing the risk of exertional heatstroke (EHS). Our theoretical framework encompasses two key components: the self‐determination theory of motivation (Deci & Ryan, 2000; Ryan & Patrick, 2009) and the concept of body awareness (Mehling et al., 2009). At its core, our hypothesis posits that EHS may arise from a disruption in the cost–benefit trade‐off associated with prolonged physical activity (Figure 1). Specifically, we suggest that EHS could be the result of an overvaluation of the benefits linked to physical activity due to excessive motivation to succeed; simultaneously, there may be an undervaluation of the physiological costs associated with effort exertion, stemming from low interoceptive awareness, that is, disrupted processing of signals about the body's internal state. To test our theoretical model, we conducted a study in a cohort of subjects, distinguishing between those with a history of exertional heatstroke (cases) and those without (controls), utilizing self‐report measures of motivation and interoceptive awareness.

The primary observation reveals that cases reported significantly lower interoceptive awareness compared to controls across the dimensions of Body listening, Attention regulation, Emotional awareness, Self‐regulation, and Noticing, as assessed through the MAIA questionnaire (Mehling et al., 2012; Willem et al., 2021). The diminished scores on the Body Listening, Attention Regulation, and Self‐Regulation subscales suggest that individuals with a history of EHS may encounter challenges actively and consistently listening to their body to gain ongoing insights into their body's internal state. In the context of physical activity, this could signify a potential difficulty in detecting physiological signs of exhaustion (e.g., hyperthermia, tachycardia, and tachypnea) that may prevent individuals from self‐regulating their physical effort, thus increasing the risk of EHS. Moreover, the low score on the Noticing subscale indicates that individuals with a history of EHS, while potentially able to sense some body signals, struggle to differentiate between negative body sensations (e.g., warning body signs of EHS) and sensations associated with physical effort in safe conditions. The reduced score on the Emotional Awareness subscale, reflecting difficulty attributing specific physical sensations to physiological manifestations of emotions (Mehling et al., 2012; Willem et al., 2021), suggests that individuals with a history of EHS may be less responsive to negative stimuli both within their bodies and in the external environment. Taken together, these findings may pave the way for the development of novel prevention strategies aimed at reducing the risk of EHS. Specifically, it is conceivable that interventions focusing on enhancing the cognitive process of interoceptive awareness could play a crucial role in mitigating the risk of EHS. By training individuals to better perceive and interpret body sensations, such programs have the potential to foster heightened interoceptive awareness, which in turn may empower individuals to implement more effective self‐regulation strategies during physical activity, thereby reducing the likelihood of EHS occurrences. Moreover, targeted health‐education messages for military personnel, athletes, and the general public can be developed from these preliminary findings. Such messages should emphasize improving active attention to one's bodily signals and learning individualized warning signs. Elite athletes—who often cultivate advanced body‐listening skills through high‐level training and mental rehearsal—illustrate how this capacity can be strengthened (Wallman‐Jones et al., 2021). Translating these athlete‐tailored strategies to broader populations could significantly bolster prevention efforts for EHS.

The second main observation is that cases demonstrated a less developed trait mindfulness compared to controls, as assessed with the FMI (Trousselard et al., 2010; Walach et al., 2006). It is pertinent to note that engaging in mindfulness practice has been demonstrated to foster the development of trait mindfulness (Brown & Ryan, 2003; Kiken et al., 2015) and enhance body awareness by training the mind to pay attention to body sensations (e.g., through the body scan exercise) (Treves et al., 2019). These findings suggest that a mindfulness‐based program, designed to train individuals to access their body sensations deliberately and continuously, may be a promising candidate as a prevention strategy to mitigate the risk of EHS. It is important to emphasize that only studies manipulating mindfulness (e.g., through mindfulness intervention) will offer conclusive evidence regarding whether mindfulness practice causally reduces the risk of EHS. We recognize that conducting such studies poses a significant methodological challenge because of the perceived rarity of EHS events (Epstein & Yanovich, 2019; Stearns et al., 2017).

The final observation in our study reveals that cases did not exhibit differences from controls concerning motivational trait (i.e., global motivation), as assessed by the GMS (Vallerand et al., 1992). Notably, the use of Bayesian statistical tests allowed us to provide moderate to strong evidence in favor of the absence of difference between cases and controls, rather than a simple declaration that the null hypothesis cannot be statistically rejected (Rouder et al., 2009; Wagenmakers et al., 2018). Although this finding is confined to the self‐report global motivation measure (see the limitations section below), it contributes to a psychometric argument derived from the GMS against a key assumption in our theoretical model, specifically that EHS could arise from an overmotivation to succeed (Figure 1). At present, it remains uncertain whether the lack of association observed between global motivation and history of EHS is pertinent to refine our model in suggesting that the factor motivation may not be relevant to inform the risk of EHS, or whether it reflects intrinsic limitations of self‐report measures (e.g., limitations of introspection and social‐desirability biases; (Baumeister et al., 2007)) to capture the cognitive construct of motivation.

Our model suggest the relevance of a broader construct of “body‐related self‐regulatory competence”, which integrates three key elements: (1) the ability to accurately sense internal signals (interoceptive awareness), (2) the capacity to interpret and respond to those signals through appropriate self‐regulation strategies, and (3) the health‐literacy skills needed to understand the significance of bodily warning signs (Fazekas, Avian, et al., 2022; Fazekas, Linder, et al., 2022). Our current findings document differences only at the first level—accurate sensing of internal signals (interoceptive awareness). However, dysregulation at subsequent levels—namely, interpreting bodily cues correctly and enacting appropriate self‐regulation—may also contribute to EHS risk. Although the MAIA captures multiple facets of interoceptive awareness (including a “Self‐Regulation” subscale; (Mehling et al., 2012; Willem et al., 2021)), it does not assess behavioral adjustment or health‐literacy skills. Future research should therefore employ ecological momentary assessment of effort adjustments and performance‐based paradigms that directly link signal detection to behavioral change, alongside instruments assessing health literacy, to test these additional regulatory levels and their cumulative impact on EHS susceptibility.

Moreover, EHS can also result from acute impairments of consciousness that bypass any cost–benefit evaluation. Heat‐induced dizziness, confusion, or cognitive slowing can immediately degrade one's ability to perceive and interpret interoceptive signals, as well as to enact self‐regulatory behaviors and sound decision‐making. While our current self‐report measures at rest reveal baseline differences between cases and controls, we anticipate these impairments would become even more pronounced under heat or exertion conditions. In such situations, individuals—even those with intact trait motivation and self‐regulatory competence—may be unable to recognize or respond to warning signs in real time. We therefore propose that future studies integrate both resting and real‐time assessments of cognitive function and conscious state (e.g., brief cognitive tasks during exertion) alongside trait measures, to capture the full spectrum of chronic vulnerabilities and acute cognitive disruptions that contribute to EHS.

### 4.2 Limits of the theoretical model and its preliminary testing

For simplicity, our cognitive model of EHS only considers the trait component of motivation that refers to the global motivational orientation of individuals at the personality level. According to the hierarchical model proposed by Vallerand (2007), self‐determined motivation can be described at additional levels of generality. The lowest level of generality corresponds to situational motivation, which pertains to the motivation experienced by an individual toward a given activity at a specific point in time (Vallerand, 2007). In our work, we did not measure the situational motivation because psychometric data were collected outside any context of physical activity. Our data show that self‐reported global motivation (trait motivation) does not help differentiate subjects with a history of EHS from healthy controls. This suggests that limiting assessment of the motivational factor to its last level of generality (i.e., the personality level) might not be informative about the risk of EHS. Future studies are encouraged to investigate the situational motivation as a potential alternative factor that could influence the risk of EHS. It could be suggested that normal (or low) global motivation combined with high situational motivation could ultimately result in a high level of self‐determined motivation. In other words, motivational factors from different levels of generality could potentially have a cumulative effect on how individuals are engaged in an activity. To test this hypothesis, experimental settings need to include measurements of both trait motivation and situational motivation, or experimentally manipulate the situational motivation of the participant by using an incentive motivation paradigm (Pessiglione et al., 2007). Classically, the measurement of situational motivation relies on self‐report instruments, such as the Situational Motivation Scale (Clancy et al., 2017; Guay et al., 2000). Yet, self‐report instruments are often criticized because they may be vulnerable to limitations of introspection and social‐desirability biases, and are potentially limited by an individual's unwillingness or inability to report their veridical cognitive state (Baumeister et al., 2007). We argue that even self‐report instruments provide valuable information and are particularly attractive for field research, they should not be considered in isolation in future cognitive research into EHS. Interestingly, some works combining behavioral measures and neurocomputational models of motivation have opened promising opportunities to address the aforementioned issue related to self‐report questionnaires. For instance, in the incentive motivation task developed by Pessiglione et al. (2007), behavioral measures (e.g., the peak of force with which the participant squeezes the power grip) can be modeled as functions that approximate the solutions of an optimal motor‐control model (which maximizes the cost/benefit tradeoff) at the individual level (Le Bouc et al., 2016; Pessiglione et al., 2007). Such a neurocomputational approach has the advantage of providing motivation‐related measures (e.g., the parameter of expected reward) that are not contaminated by individual differences in other cognitive components (e.g., emotional thoughts, changes in attention, etc.). Therefore, we encourage future studies investigating the motivational underpinnings of EHS to use this neurocomputational approach that has great potential to enhance the process of relating differences (in behavior and neural processes) between healthy subjects and individuals who are at risk of EHS.

Regarding the other main component of our model that refers to interoceptive body awareness, it has been formalized as a multifaceted cognitive process that can be interrogated with complementary methods (Garfinkel et al., 2015; Khalsa & Lapidus, 2016), including self‐report instruments and objective measurements (e.g., behavioral test or biomarker). Objective measurements of interoceptive body awareness mainly focus on cardiac interoception that refers to the process of sensing, storing, and representing information about the state of the cardiovascular system (Garfinkel et al., 2015; Khalsa & Lapidus, 2016). These measurements are mostly performed under conditions of physiological rest, that is, without any significant experimentally induced cardiovascular manipulation, which raises questions about their potential relevance to inform interoceptive dysfunction in the context of EHS. Indeed, physical effort is characterized by strong, continuous perturbations that affect the cardiovascular system (e.g., increased heart rate and arterial pressure). Interestingly, the pharmacological manipulation of cardiac arousal (via the administration of isoproterenol that modulates sympathetic nervous system) may provide an attractive experimental framework because it has the advantage of a maskable manipulation of arousal (including placebo condition) that allows for measurements of responding bias (Khalsa et al., 2009). Besides behavioral tests that provide an indirect output of interoceptive signal processing, the neural bases of cardiac interoception can be investigated by probing brain activity in response to cardiac signals; for example, the Heartbeat Evoked Potential, which refers to evoked changes in brain activity (measured using magnetoencephalography, electroencephalography, or intracranial neural recordings) that occurs after a heartbeat, has been proposed as a neurophysiological marker of interoceptive function/dysfunction (Coll et al., 2021; Park & Blanke, 2019).

To summarize, future studies are encouraged to pursue the development and validation of a cognitive model for EHS using a neurocomputational approach of motivation with the incentive motivation paradigm (Pessiglione et al., 2007), and objective measurements of interoceptive body awareness (e.g., heartbeat perception task (Brener & Ring, 2016) or analysis of the heartbeat evoked potential (Coll et al., 2021; Park & Blanke, 2019)) based on a paradigm involving pharmacological manipulation of cardiac arousal (Khalsa et al., 2009).

## 5 CONCLUSION

In this study, we proposed and provided preliminary empirical support for a cognitive model of exertional heatstroke (EHS). Individuals with a history of EHS exhibited significantly diminished interoceptive awareness and reduced trait mindfulness compared to controls, but did not differ in global motivation trait. These results identify low interoceptive awareness as a potential intrinsic risk factor for EHS, and suggest that cognitive neuroscience–particularly interoception research–offers a promising avenue for translational studies. Our findings also encourage future research on mindfulness‐based preventive strategies and the assessment of situational motivation as additional pathways for reducing EHS risk in athletic and military contexts.

## AUTHOR CONTRIBUTIONS

CM and PETD conceptualized the research question, collected experimental data, and wrote the paper. CV conceptualized the research question, conducted the analyses, and wrote the paper. AM, AJ, and KC conceptualized the research question and collected experimental data. MT contributed to conceptualizing the research question. All authors approved the final version of the manuscript for submission.

## PREREGISTRATION

This study was preregistered (NCT04593316).

## FUNDING INFORMATION

This study is part of a project supported by the French Military Health Service.

## CONFLICT OF INTEREST STATEMENT

The authors declare that they have no competing interests.

## ETHICS STATEMENT

This study was approved by the regional ethics committee of the Agence Régionale de Santé Occitanie (Comité de Protection des Personnes Sud‐Ouest et Outre‐Mer II, ID‐RCB: 2020‐A01967‐32) on September 29, 2020. Written informed consent was obtained from all individual participants included in the study. The study was conducted in accordance with the ethical standards of the 1964 Helsinki declaration and its later amendments.

## Supporting information


Appendix S1.

## Data Availability

We do not have permission to deposit military data in a public repository, but the French Military Health Service at dcssa-paris@sante.defense.gouv.fr can be approached for any queries related to the data used in this paper.
