# Supplementary material for: Feeling the heat: Investigating interoception and motivation as risk factors for exertional heatstroke
Source: Physiol Rep. 2025 Oct 16;13(20):e70529. doi: 10.14814/phy2.70529 (PMC12531346; doi:10.14814/phy2.70529)
Supplement: Supplementary file 1 — Appendix S1. [file PHY2-13-e70529-s001.zip › Revised_Supplement.docx]

**SUPPLEMENTARY CONTENT**

**Feeling the heat: Investigating interoception and motivation as risk factors**

**for exertional heatstroke**

Charles Verdonk ^(a,b,c)^, Camille Mellier ^(d)^, Keyne Charlot ^(e,f)^, Arnaud Jouvion ^(g)^,

Marion Trousselard ^(a,h)^, Emmanuel Sagui ^(a)^, Alexandra Malgoyre ^(d,e,f)^,

and Pierre-Emmanuel Tardo-Dino ^(e,f)^

**Authors’ affiliations**

1. French Armed Forces Biomedical Research Institute

Department of Neuroscience and cognitive science

Unit of Neurophysiology of stress

91220 Brétigny-sur-Orge, France

1. UMR VIFASOM

Université de Paris

75004 Paris, France

1. Laureate Institute for Brain Research

Tulsa, OK, USA

1. French Military Health Service Academy

75005 Paris, France

1. French Armed Forces Biomedical Research Institute

Department of Operational Environments

Unit of Physiology of exercise and physical activities in extreme conditions

91220 Brétigny-sur-Orge, France

1. Exercise Biology for Performance and Health Laboratory

University Evry-Paris Saclay

91042 Evry, France

1. French Military Teaching Hospital Laveran

13000 Marseille, France

1. École de Psychologues Praticiens

Catholic Institute of Paris

EA Religion, culture et société

75006 Paris, France

**Corresponding author:** Charles Verdonk, [verdonk.charles@gmail.com](mailto:verdonk.charles@gmail.com)

**Table of contents**

**Supplementary Introduction…………………………………………...…………………........Page 4**

**Supplementary Methods………………………………………………………………………...Page 6**

**Supplementary Tables……………………………………………………………………..........Page 7**

**Supplementary References……………………..……………………………………............Page 12**

**Supplementary Introduction**

**
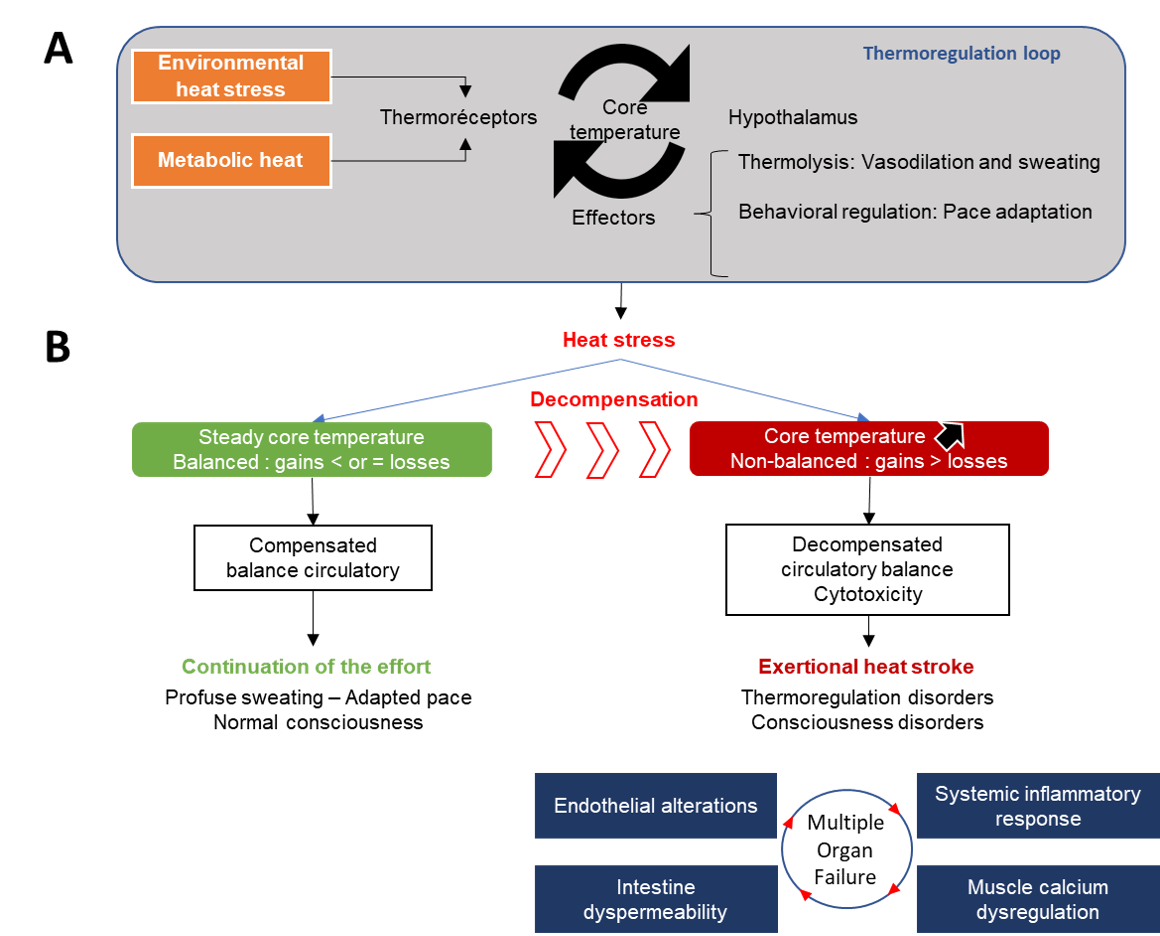
**

**Supplementary Fig. 1.** Pathogenesis of exertional heatstroke: a graphical overview of suspected physiological mechanisms. **(A) Physiological thermoregulation.** Whatever exogene (outdoor) and/or endogene (exercise metabolism), heat exposure draws a regulatory loop. Thermoreceptors inform the thermoregulatory centers (hypothalamus) that will coordinate the response of the effectors. Thus, heat dissipation is obtained by generalized subcutaneous vasodilation and sweat evaporation. The reduction of heat stress can also come from behavioral regulation including the adaptation of the pace (decrease in metabolic production) and the search for shelter and the adaptation of the clothing (decrease of the external heat load). **(B) Pathophysiology of exertional heat stroke.** The occurrence of exercise heat stroke proceeds from the transition from a compensation of heat load to a non-compensable heat stress phasis (gains greater than heat losses) occurring when the cardiac output no longer allows to provide for thermoregulation needs. This uncontrolled hyperthermia leads to cytotoxic effects and a systemic inflammatory response that can lead to multi-organ failure. In this context of decompensated circulatory balance, the pathophysiological mechanisms would be based on disorders of intestinal permeability with release of activating molecules of the inflammatory and immune system. Endothelial alterations would also be responsible for coagulopathy. The direct cytotoxic effect of the temperature increase could also induce brain alterations, particularly hypothalamic. In a certain case, mutations in the ryanodine receptor RyR 1 (a muscle receptor involved in the release of intracellular calcium during contraction) could promote the occurrence of this decompensation of thermoregulation to exercise, by the disorders of the excitation coupling contraction that they induce (Epstein and Yanovich, 2019; Laitano et al., 2019).

**Supplementary Methods**

**Power considerations**

To ensure that our analyses were nevertheless adequately powered, we conducted post hoc power analyses on significant data. For this purpose, we used the GPOWER software with the following parameters: Tails: Two; $\alpha$ error probability: 0.05; Total sample size: 94 (51 cases + 43 controls). The effect size Cohen’s d effect size was computed from the reported rank-biserial correlation from each Mann–Whitney U test as follows (Equation 1):

|  | $\mathrm{Cohen}^{'}s d=\frac{{2r}_{\mathrm{rb}}}{\sqrt{1-r_{\mathrm{rb}}^{2}}}$ | (1) |
| --- | --- | --- |

**Supplementary Tables**

**Supplementary Table 1.** A descriptive and approximate classification scheme for the interpretation of the log scale of Bayes factor BF_10_ (adapted from (Jeffreys, 1961)).

**
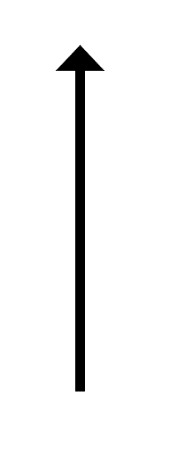

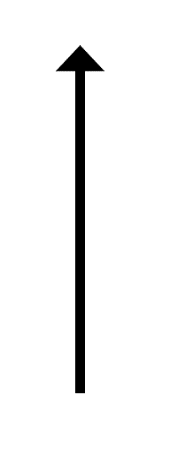
**

Growing evidence in favour of H_0_

Growing evidence in favour of H_1_

| **Log (BF_10_)** | **Interpretation** | **Symbol** |
| --- | --- | --- |
|  |  |  |
| > 2 | extreme evidence for H_1_ | H_1_**^****^** |
| [1.48 ; 2] | very strong evidence for H_1_ | H_1_**^***^** |
| [1 ; 1.48] | strong evidence for H_1_ | H_1_^**^ |
| [0.48 ; 1] | moderate evidence for H_1_ | H_1_^*^ |
| [0 ; 0.48] | anecdotal evidence for H_1_ | ns |
| 0 | no evidence | ns |
| [-0.48 ; 0] | anecdotal evidence for H_0_ | ns |
| [-1 ; -0.48] | moderate evidence for H_0_ | H_0_**^*^** |
| [-1.48 ; -1] | strong evidence for H_0_ | H_0_**^**^** |
| [-2 ; -1.48] | very strong evidence for H_0_ | H_0_**^***^** |
| < -2 | extreme evidence for H_0_ | H_0_**^****^** |

Log(BF_10_): log scale of Bayes factor BF_10_; H_1_: alternative hypothesis; ns: non-significant; H_0_: null hypothesis

**Supplementary Table 2.** Descriptive statistics for the Multidimensional Assessment of Interoceptive Awareness (MAIA) questionnaire in cases with a history of exertional heatstroke and controls.

|  | **Cases**  (n=51) | | **Controls**  (n=43) | |
| --- | --- | --- | --- | --- |
|  | M | SD | M | SD |
| Scale ***Noticing*** | 3.52 | 0.87 | 3.88 | 0.72 |
| Scale ***Not-distracting*** | 2.46 | 0.89 | 2.77 | 0.90 |
| Scale ***Not-worrying*** | 2.91 | 0.89 | 3.02 | 0.93 |
| Scale ***Attention regulation*** | 3.05 | 0.89 | 3.55 | 0.76 |
| Scale ***Emotional awareness*** | 3.44 | 0.95 | 3.88 | 0.81 |
| Scale ***Self-regulation*** | 2.97 | 1.15 | 3.50 | 0.98 |
| Scale ***Body listening*** | 2.37 | 1.10 | 3.05 | 1.10 |
| Scale ***Trusting*** | 3.80 | 0.89 | 4.11 | 0.96 |
| **Total score** | 24.53 | 4.81 | 27.76 | 4.52 |
|  |  |  |  |  |

M: mean; SD: standard deviation

**Supplementary Table 3.** Descriptive statistics for the Global Motivation Scale (GMS) questionnaire in cases with a history of exertional heatstroke and controls.

|  | **Cases**  (n=51) | | **Controls**  (n=43) | |
| --- | --- | --- | --- | --- |
|  | M | SD | M | SD |
| Scale ***IM to know*** | 21.61 | 4.51 | 22.54 | 3.89 |
| Scale ***IM to accomplishment*** | 22.25 | 4.15 | 22.07 | 4.73 |
| Scale ***IM to stimulation*** | 20.47 | 4.70 | 20.56 | 5.04 |
| Scale ***Identified regulation*** | 21.92 | 3.74 | 21.58 | 3.87 |
| Scale ***Introjected regulation*** | 18.18 | 5.10 | 17.93 | 5.54 |
| Scale ***External regulation*** | 18.96 | 5.23 | 17.33 | 5.96 |
| Scale ***Amotivation*** | 11.43 | 4.08 | 14.51 | 5.59 |
|  |  |  |  |  |

M: mean; SD: standard deviation; IM: intrinsic motivation

**Supplementary Table 4.** Descriptive statistics for the Freiburg Mindfulness Inventory in cases with a history of exertional heatstroke and controls.

|  | **Cases**  (n=51) | | **Controls**  (n=43) | |
| --- | --- | --- | --- | --- |
|  | M | SD | M | SD |
| Scale ***Presence*** | 18.69 | 3.02 | 20.30 | 2.67 |
| Scale ***Acceptation*** | 21.57 | 3.79 | 23.77 | 3.70 |
| **Total score** | 40.25 | 6.18 | 44.07 | 5.34 |
|  |  |  |  |  |

M: mean; SD: standard deviation

**Supplementary Table 5.** Post hoc power analysis for interoceptive and motivation dimensions showing significant group differences.

|  | rbs  (midpoint of CI) | Cohen’s d | Achieved Power (1-β) |
| --- | --- | --- | --- |
| Scale ***Noticing*** | 0.22 | 0.45 | 0.79 |
| Scale ***Attention regulation*** | 0.32 | 0.68 | 0.93 |
| Scale ***Emotional awareness*** | 0.29 | 0.60 | 0.85 |
| Scale ***Self-regulation*** | 0.28 | 0.57 | 0.82 |
| Scale ***Body listening*** | 0.33 | 0.70 | 0.94 |
| Scale ***Amotivation*** | 0.32 | 0.68 | 0.93 |
|  |  |  |  |

Rbs: rank-biserial correlation; CI: confidence interval;

**Supplementary references**

Epstein, Y., and Yanovich, R. (2019). Heatstroke. *New England Journal of Medicine* 380(25)**,** 2449-2459.

Jeffreys, H. (1961). *Theory of probability.*

Laitano, O., Leon, L.R., Roberts, W.O., and Sawka, M.N. (2019). Controversies in exertional heat stroke diagnosis, prevention, and treatment. *Journal of Applied Physiology* 127(5)**,** 1338-1348.
