# Supplementary material for: Feeling the heat: Investigating interoception and motivation as risk factors for exertional heatstroke
Source: Physiol Rep. 2025 Oct 16;13(20):e70529. doi: 10.14814/phy2.70529 (PMC12531346; doi:10.14814/phy2.70529)
Supplement: Supplementary file 1 — Appendix S1. [file PHY2-13-e70529-s001.zip › Revised_Supplement.pdf]

SUPPLEMENTARY CONTENT

Feeling the heat: Investigating interoception and motivation as risk factors for exertional heatstroke

Charles Verdonk [a,b,c], Camille Mellier [d], Keyne Charlot [e,f], Arnaud Jouvion [g], Marion Trousselard [a,h], Emmanuel Sagui [a], Alexandra Malgoyre [d,e,f], and Pierre-Emmanuel Tardo-Dino [e,f]

Authors' affiliations

[a] French Armed Forces Biomedical Research Institute
Department of Neuroscience and cognitive science
Unit of Neurophysiology of stress
91220 Brétigny-sur-Orge, France

[b] UMR VIFASOM
Université de Paris
75004 Paris, France

[c] Laureate Institute for Brain Research
Tulsa, OK, USA

[d] French Military Health Service Academy
75005 Paris, France

[e] French Armed Forces Biomedical Research Institute
Department of Operational Environments
Unit of Physiology of exercise and physical activities in extreme conditions
91220 Brétigny-sur-Orge, France

[f] Exercise Biology for Performance and Health Laboratory
University Evry-Paris Saclay
91042 Evry, France

[g] French Military Teaching Hospital Laveran
13000 Marseille, France

[h] École de Psychologues Praticiens
Catholic Institute of Paris
EA Religion, culture et société
75006 Paris, France

Corresponding author: Charles Verdonk, verdonk.charles@gmail.com

Table of contents

Supplementary Introduction……………………………………...………………….........Page 4
Supplementary Methods……………………………………………………………...Page 6
Supplementary Tables…………………………………………………………….........Page 7
Supplementary References…………………...……………………………………...........Page 12

Supplementary Introduction

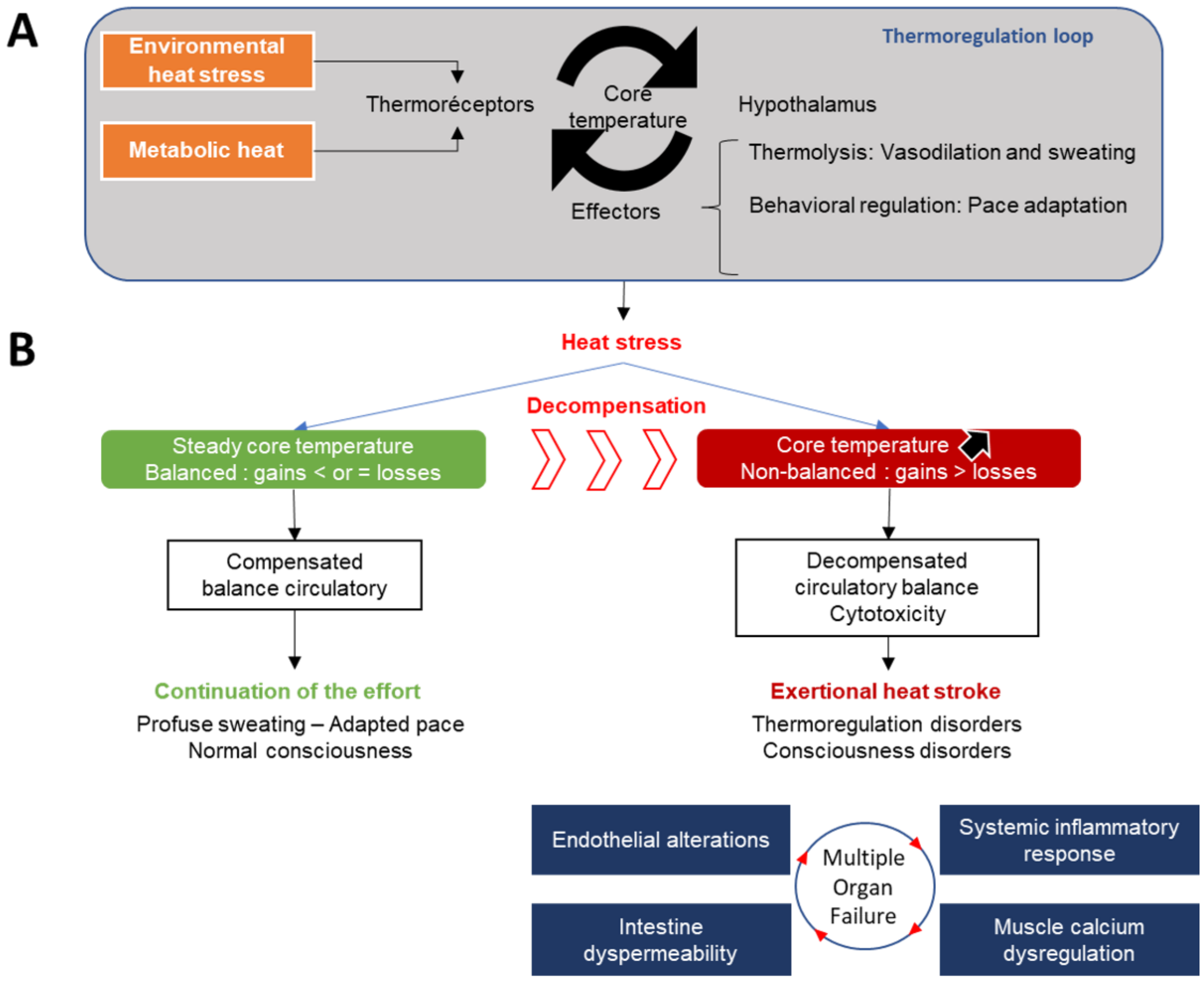

Supplementary Fig. 1. Pathogenesis of exertional heatstroke: a graphical overview of suspected physiological mechanisms. **(A) Physiological thermoregulation.** Whatever exogene (outdoor) and/or endogene (exercise metabolism), heat exposure draws a regulatory loop. Thermoreceptors inform the thermoregulatory centers (hypothalamus) that will coordinate the response of the effectors. Thus, heat dissipation is obtained by generalized subcutaneous vasodilation and sweat evaporation. The reduction of heat stress can also come from behavioral regulation including the adaptation of the pace (decrease in metabolic production) and the search for shelter and the adaptation of the clothing (decrease of the external heat load). **(B) Pathophysiology of exertional heat stroke.** The occurrence of exercise heat stroke proceeds from the transition from a compensation of heat load to a non-compensable heat stress phasis

(gains greater than heat losses) occurring when the cardiac output no longer allows to provide for thermoregulation needs. This uncontrolled hyperthermia leads to cytotoxic effects and a systemic inflammatory response that can lead to multi-organ failure. In this context of decompensated circulatory balance, the pathophysiological mechanisms would be based on disorders of intestinal permeability with release of activating molecules of the inflammatory and immune system. Endothelial alterations would also be responsible for coagulopathy. The direct cytotoxic effect of the temperature increase could also induce brain alterations, particularly hypothalamic. In a certain case, mutations in the ryanodine receptor RyR 1 (a muscle receptor involved in the release of intracellular calcium during contraction) could promote the occurrence of this decompensation of thermoregulation to exercise, by the disorders of the excitation coupling contraction that they induce (Epstein and Yanovich, 2019; Laitano et al., 2019).

Supplementary Methods

Power considerations

To ensure that our analyses were nevertheless adequately powered, we conducted post hoc power analyses on significant data. For this purpose, we used the GPOWER software with the following parameters: Tails: Two; α error probability: 0.05; Total sample size: 94 (51 cases + 43 controls). The effect size Cohen's d effect size was computed from the reported rank-biserial correlation from each Mann–Whitney U test as follows (Equation 1):

$$\text{Cohen's d} = \frac{2\mathrm{r}_{\mathrm{rb}}}{\sqrt{1 - \mathrm{r}_{\mathrm{rb}}^{2}}} \qquad (1)$$

Supplementary Tables

Supplementary Table 1. A descriptive and approximate classification scheme for the interpretation of the log scale of Bayes factor BF_{10} (adapted from (Jeffreys, 1961)).

	**Log (BF_{10})**	**Interpretation**	**Symbol**
Growing evidence in favour of H_1 ↑	> 2	extreme evidence for H_1	H_1^{****}
	[1.48 ; 2]	very strong evidence for H_1	H_1^{***}
	[1 ; 1.48]	strong evidence for H_1	H_1^{**}
	[0.48 ; 1]	moderate evidence for H_1	H_1^{*}
	[0 ; 0.48]	anecdotal evidence for H_1	ns
	0	no evidence	ns
Growing evidence in favour of H_0 ↓	[-0.48 ; 0]	anecdotal evidence for H_0	ns
	[-1 ; -0.48]	moderate evidence for H_0	H_0^{*}
	[-1.48 ; -1]	strong evidence for H_0	H_0^{**}
	[-2 ; -1.48]	very strong evidence for H_0	H_0^{***}
	< -2	extreme evidence for H_0	H_0^{****}

Log(BF_{10}): log scale of Bayes factor BF_{10}; H_1: alternative hypothesis; ns: non-significant; H_0: null hypothesis

Supplementary Table 2. Descriptive statistics for the Multidimensional Assessment of Interoceptive Awareness (MAIA) questionnaire in cases with a history of exertional heatstroke and controls.

	**Cases** (n=51)		**Controls** (n=43)	
	M	SD	M	SD
Scale ***Noticing***	3.52	0.87	3.88	0.72
Scale ***Not-distracting***	2.46	0.89	2.77	0.90
Scale ***Not-worrying***	2.91	0.89	3.02	0.93
Scale ***Attention regulation***	3.05	0.89	3.55	0.76
Scale ***Emotional awareness***	3.44	0.95	3.88	0.81
Scale ***Self-regulation***	2.97	1.15	3.50	0.98
Scale ***Body listening***	2.37	1.10	3.05	1.10
Scale ***Trusting***	3.80	0.89	4.11	0.96
**Total score**	24.53	4.81	27.76	4.52

M: mean; SD: standard deviation

Supplementary Table 3. Descriptive statistics for the Global Motivation Scale (GMS) questionnaire in cases with a history of exertional heatstroke and controls.

	**Cases** (n=51)		**Controls** (n=43)	
	M	SD	M	SD
Scale ***IM to know***	21.61	4.51	22.54	3.89
Scale ***IM to accomplishment***	22.25	4.15	22.07	4.73
Scale ***IM to stimulation***	20.47	4.70	20.56	5.04
Scale ***Identified regulation***	21.92	3.74	21.58	3.87
Scale ***Introjected regulation***	18.18	5.10	17.93	5.54
Scale ***External regulation***	18.96	5.23	17.33	5.96
Scale ***Amotivation***	11.43	4.08	14.51	5.59

M: mean; SD: standard deviation; IM: intrinsic motivation

Supplementary Table 4. Descriptive statistics for the Freiburg Mindfulness Inventory in cases with a history of exertional heatstroke and controls.

	**Cases** (n=51)		**Controls** (n=43)	
	M	SD	M	SD
Scale ***Presence***	18.69	3.02	20.30	2.67
Scale ***Acceptation***	21.57	3.79	23.77	3.70
**Total score**	40.25	6.18	44.07	5.34

M: mean; SD: standard deviation

Supplementary Table 5. Post hoc power analysis for interoceptive and motivation dimensions showing significant group differences.

	rbs (midpoint of CI)	Cohen's d	Achieved Power (1-β)
Scale ***Noticing***	0.22	0.45	0.79
Scale ***Attention regulation***	0.32	0.68	0.93
Scale ***Emotional awareness***	0.29	0.60	0.85
Scale ***Self-regulation***	0.28	0.57	0.82
Scale ***Body listening***	0.33	0.70	0.94
Scale ***Amotivation***	0.32	0.68	0.93

Rbs: rank-biserial correlation; CI: confidence interval;

Supplementary references

Epstein, Y., and Yanovich, R. (2019). Heatstroke. *New England Journal of Medicine* 380(25)**,** 2449-2459.

Jeffreys, H. (1961). *Theory of probability.*

Laitano, O., Leon, L.R., Roberts, W.O., and Sawka, M.N. (2019). Controversies in exertional heat stroke diagnosis, prevention, and treatment. *Journal of Applied Physiology* 127(5)**,** 1338-1348.
